# Integrating the social determinants of health into graduate medical education training: a scoping review

**DOI:** 10.1186/s12909-024-05394-2

**Published:** 2024-05-23

**Authors:** Nehal Nour, David Onchonga, Siobhan Neville, Patrick O’Donnell, Mohamed Elhassan Abdalla

**Affiliations:** Faculty of Education & Health Services, School of Medicine, University of Limerick, Garraun, Castletroy, Co. Limerick, V94 T9PX Ireland

**Keywords:** Social determinants of health, Graduate medical education, Medical trainees, Training program

## Abstract

**Background:**

The social determinants of health (SDH) play a key role in the health of individuals, communities, and populations. Academic institutions and clinical licensing bodies increasingly recognize the need for healthcare professionals to understand the importance of considering the SDH to engage with patients and manage their care effectively. However, incorporating relevant skills, knowledge, and attitudes relating to the SDH into curricula must be more consistent. This scoping review explores the integration of the SDH into graduate medical education training programs.

**Methods:**

A systematic search was performed of PubMed, Ovid MEDLINE, ERIC, and Scopus databases for articles published between January 2010 and March 2023. A scoping review methodology was employed, and articles related to training in medical or surgical specialties for registrars and residents were included. Pilot programs, non-SDH-related programs, and studies published in languages other than English were excluded.

**Results:**

The initial search produced 829 articles after removing duplicates. The total number of articles included in the review was 24. Most articles were from developed countries such as the USA (22), one from Canada, and only one from a low- and middle-income country, Kenya. The most highly represented discipline was pediatrics. Five papers explored the inclusion of SDH in internal medicine training, with the remaining articles covering family medicine, obstetrics, gynecology, or a combination of disciplines. Longitudinal programs are the most effective and frequently employed educational method regarding SDH in graduate training. Most programs utilize combined teaching methods and rely on participant surveys to evaluate their curriculum.

**Conclusion:**

Applying standardized educational and evaluation strategies for SDH training programs can pose a challenge due to the diversity of the techniques reported in the literature. Exploring the most effective educational strategy in delivering these concepts and evaluating the downstream impacts on patient care, particularly in surgical and non-clinical specialties and low- and middle-income countries, can be essential in integrating and creating a sustainable healthcare force.

## Introduction

The World Health Organization (WHO) defines the social determinants of health (SDH) as “the conditions in which people are born, grow, live, work, and age, that affect a wide range of health and quality of life outcomes.” These conditions are brought about by the nature in which resources, finances, and power are distributed locally, nationally, and globally and may include economic policies and systems, development agendas, social norms, social policies, and political systems [1]. SDH can have a significant impact on individual and population health. Studies have demonstrated that marginalized individuals and communities suffering discrimination have noticeably poorer health outcomes [2]..

There has been a clarion call to integrate SDH concepts for doctors seeking postgraduate training to equip future healthcare professionals with the appropriate competencies to tackle SDH-related factors at the patient and community level [3–5]. A critical understanding of the causes and impacts of SDH by doctors is needed to provide effective healthcare while offering adequate stewardship of limited resources and promoting health equity of the populations they serve [6]. Orienting medical training towards SDH is a significant step to equip physicians with the understanding, proficiencies, and attitudes needed to begin to address health inequalities [7].

Medical education regarding the SDH is crucial for future medical practitioners [8]. Besides potentially enhancing health outcomes for individual patients, physicians tackling these disparities will adopt the initiatives calling for changes to influence population and community health [9–11]. Thus, understanding social determinants of health requires a perspective shift for graduate learners, with the desired educational outcome being transformative learning [12, 13].

Despite a growing understanding of the importance of integrating SDH into health professional curricula, the optimum approach to incorporating SDH teaching into undergraduate and graduate training curricula has yet to be clarified. A comprehensive guide for SDH teaching strategies would promote consistency in graduate training. A previous scoping review explored the inclusion of SDH in undergraduate medical curricula. The study highlighted the benefits of longitudinal curricula with community involvement in developing retainable knowledge and skills regarding SDH for medical students [14]. In 2019, a scoping review exploring the graduate curriculum interventions focused on SDH objectives concluded the insufficient physician training regarding SDH covers Canada only [15]..

This scoping review was performed to explore the extent of integration of SDH in graduate medical education curricula globally. The study objective was to explore the structure, content, training strategies, and evaluation methods used in incorporating SDH into training qualified doctors seeking higher medical training.

## Methods

The scoping review was performed by searching four relevant databases – PubMed, Ovid MEDLINE, ERIC, and Scopus. The process was undertaken by standard scoping review methodology, including identifying the research question, identifying relevant studies, selecting studies, charting the data, and collating, summarizing, and reporting the results [16].

i. *Formulation of the research question*

All authors formulated the research question, guided by the WHO’s definition of social determinants of health [1]. The overall question: What has been published on the topic of the integration of SDH into graduate medical education curricula? Specifically, the research question focused on the content of the SDH teaching in the graduate medical curriculum, their presentation, teaching strategies, and program evaluation. It aimed to identify any gaps in the available literature to guide future research.

ii. *Identification of relevant studies, including the data sources and search strategy*

**Fig. 1:**
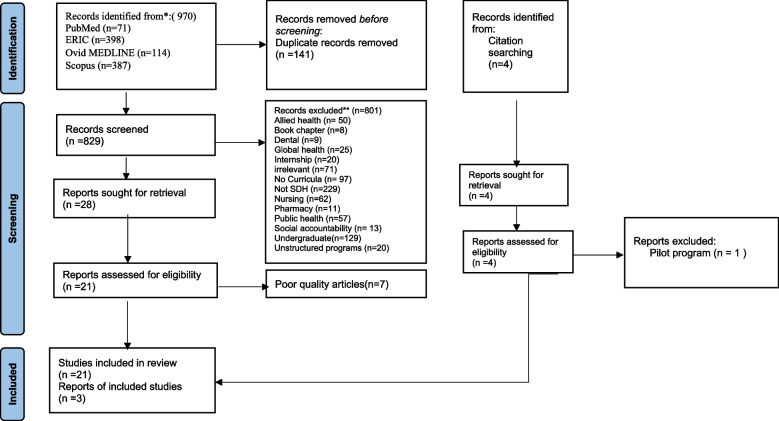
PRISMA flow diagram for the systematic scoping review of the SDH post-graduate training program *Consider, if feasible to do so, reporting the number of records identified from each database or register searched (rather than the total number across all databases/registers). **If automation tools were used, indicate how many records were excluded by a human and how many were excluded by automation tools. *From:* Page MJ, McKenzie JE, Bossuyt PM, Boutron I, Hoffmann TC, Mulrow CD, et al. The PRISMA 2020 statement: an updated guideline for reporting systematic reviews. BMJ 2021;372:n71. doi: 10.1136/bmj.n71. For more information, visit: (38)

Authors searched PubMed, Ovid MEDLINE, ERIC, and Scopus in March 2023. Individual search strategies were developed for each database, and searches were run for each database (Table 1). The search strategy was comprehensive to capture the diversity of the potential SDH integrated into the graduate medical education curricula. PRISMA-ScR guidelines [17, 18] were followed, as illustrated in (Fig. 1). The study population consisted of medical professionals (doctors) in any discipline undertaking postgraduate training, including specialty trainees, residents, fellows, and registrars; the concept was the content of the curriculum used for teaching the SDH, with the context being graduate medical schools and training health facilities and institutes globally.

iii. *Identifying relevant studies*

**Table 1:** Search Strategy for the Databases regarding the SDH Postgraduate Training

Ovid MEDLINE(R) ALL < 1946 to March 10, 2023>	
1. Social determinants of health.mp. or exp. *"Social Determinants of Health”/13,808	
2. exp. *General Practitioners/ or registrar.mp. or exp. *Medical Staff, Hospital/27288	
3. Residency.mp. or exp. *"Internship and Residency”/74,897	
4. 2 or 3 100,551	
5. “Clinical competency”.mp. or exp. *Clinical Competence/50511	
6. Curriculum.mp. or exp. *Curriculum/117182	
7. exp. *education, professional/ or exp. *clinical clerkship/ or education, continuing/ or exp. *education, dental/ or exp. *education, graduate/ or exp. *education, medical/ or exp. *education, medical, continuing/ or exp. *education, medical, graduate/247393	
8. 5 or 6 or 7 333,122	
9. training.mp. 569,120	
10. 8 or 9 812,932	
11. 1 and 4 167	
12. 10 and 11 114	
**PubMed (covered till March 2023)**	
1. “Social Determinants of Health”[Mesh]	
2. “Social Determinants of Health”[Title/Abstract] OR SDH[Title/Abstract]	
3. #1 and #2	
4. Residency [Text Word] OR Training [Text Word]	
5. #3 and #4	
6 .curriculum [Text Word] OR curricula [Text Word] OR teaching [Text Word]	
7. #5 and #6	
8. (((“Social Determinants of Health”[Mesh]) and (“Social Determinants of Health”[Title/Abstract] OR SDH[Title/Abstract])) and (Residency[Text Word] OR Training[Text Word])) and (curriculum[Text Word] OR curricula[Text Word] OR teaching[Text Word])	
**Scopus: (covered till March 2023)**	
(TITLE-ABS-KEY (*“Social determinants of health”* OR *SDH*) AND KEY (*training* OR *learning* OR *teaching* OR *“medical education”* OR *“medical training”*) AND KEY (*specialist* OR *registrar* OR *residency*) OR KEY (*curriculum* OR *curricula***)**) AND (EXCLUDE (PUBYEAR, *2008*) OR EXCLUDE (PUBYEAR, *2007*) OR EXCLUDE (PUBYEAR, *2006*) OR EXCLUDE (PUBYEAR, *2005*)	
**ERIC: (covered till March 2023)**	
((“Social determinants of health” or SDH) AND (Curriculum* OR teaching* OR learning* OR competency*) OR (“Clinical Competency” OR “medical education”) AND (specialist OR registrar OR residency OR internship OR fellowship))	
Entered date: 2010–2023

Two authors (DO, NN) reviewed relevant articles after the initial removal of duplicates by exporting the references to Mendeley Reference Manager [19]; articles were analyzed using Rayyan [20], an online software that helps with a blinded screening of articles. Two authors (DO, NN) then independently screened the titles and abstracts without limiting the articles’ publication dates, population, and study locations. The remaining articles underwent full-text screening, and a third author was called to arbitrate where there were differences in screening outcomes.

iv. *Inclusion and exclusion criteria*

Articles were deemed eligible for inclusion if they focused on graduate SDH curricula, including fellows, registrars, trainees, and residents. Studies had to contain structural curricula to qualify for inclusion. Articles published in English between January 2012 and March 2023 were included in the current study. If the program did not intend to integrate the SDH in graduate medical education or did not indicate a mechanism for evaluating the curriculum, they were excluded from this review. Also, the following exclusion criteria were applied: undergraduate programs, reports, systematic reviews, pilot programs, unstructured programs, programs not focusing on SDH teaching, programs not in English, internship studies, and studies that focused on allied health programs such as nursing, public health, global health, dentistry, and pharmacy.

xxii. *Charting the data*

The main characteristics of each graduate SDH medical curriculum were detailed, including the discipline integrating the program, the program title, length, educational methods, teaching concepts, and methods of curriculum evaluation. In this stage, data from the selected articles were extracted to a Microsoft Excel sheet, and key information about the authors and year of publication was included.

vi. *Quality assessment tool.*

Two reviewers (DO, NN) performed an independent quality assessment for each article. The Medical Education Research Study Quality Instrument (MERSQI) [21] was selected for quality appraisal of the included articles. The appraisal tools assessed the articles over six domains – study design, sampling, type of data, validity of the evaluation, data synthesis, and outcome. All the included articles had a score of 9 and above, which is acceptable.

## Results

The original search yielded 970 articles. A total of 141 duplicates were removed. In the initial title and abstract screening step, 829 articles were examined. A further 801 articles were removed upon applying exclusion criteria. The exclusion criteria were: unrelated to SDH (*n* = 229), associated with undergraduate curricula (*n* = 129), not curriculum-based (*n* = 97), irrelevant (*n* = 71), nursing curricula (*n* = 62), related to public health and disease prevention (*n* = 57), allied health curricula (*n* = 50), considered with global health and elimination of global issues (*n* = 25), internship (*n* = 20), unstructured programs (*n* = 20), social accountability (*n* = 13), pharmacy curricula (*n* = 11), dentistry curricula (*n* = 9) and book chapter (*n* = 8).

Only 28 articles met the inclusion criteria. The next step was a full examination of the 28 articles that met the inclusion criteria and whose focus was oriented toward the contents of the SDH in graduate medical education. At this point, we removed seven articles as they did not meet the quality assessment criteria.

A total of 21 articles met the inclusion criteria and were included in the review. A hand search through the references of the included articles yielded another four studies; three were deemed eligible for inclusion, and one pilot program was excluded. The final number of articles included in the review was 24.

### Summary of the graduate SDH training programs

Of the 24 programs included in the current scoping review, 22 were from graduate residency programs in the United States of America(USA), one from Canada, and one from a residency program in Kenya. Almost 50% (*n* = 12) of the articles were based on pediatric graduate curricula, while nearly 21% (*n* = 5) were from internal medicine programs, as indicated in Table 2.

**Table 2:** Number of articles in each Post-graduate Speciality Program

S/No	Post-graduate specialty program	Number of articles
1	Paediatrics	12
2	Internal medicine	5
3	Family medicine	3
4	Family medicine and Internal medicine	1
5	Family medicine and psychiatric medicine	1
6	Family medicine, emergency medicine and internal medicine	1
7	Obstetrics and gynaecology	1
	Total	24

### Structure and duration of the postgraduate SDH training

As Table 3 illustrates, of the 24 articles analyzed, the duration of the program relating to SDH varied. Twelve programs had longitudinal modules, spanning one to 3 years in the postgraduate medical residency [22–33], while five other programs spanned two to 9 months in the postgraduate medical residency [34–38]. Seven programs took between 2 weeks and 6 weeks [39–43, 43, 44], while the shortest program involved three online simulations; each simulation is 4 hours (one-half day) and completed during a module on advocacy [45].

**Table 3:** Study summary of SDH training programs

	Author	Medical Institution	Program Title	Evaluation Method (if applicable)	Targeted Trainees	Participants	Program Content	Structure
1	Bradley et al., 2021	The Veterans Health Administration Hospital, Vermont, United States.	Healing Through History	Post program focus group interviews	Internal medicine residents	46 completed program 12 participated in focus group	Listening to and co-producing, and reflection on a single in-depth patient story during the first year of residency to generate educational and clinical value that would be integrated with other learning experiences.	Two- half day sessions to identify and interview patients, followed by 90 minutes FGD, 6 weeks from the experience.
2	Daya et al.,2021	San Francisco Tertiary Hospital, United States.	Advocacy in Action	Thematic analysis of written reflections	Internal medicine residents	66 completed program 49 participated in reflection	Patient communication skills, patient education and counselling skills within their social context and collaborative care skills.	Two month
3	Nelligan et al.,2016	Moi University Faculty of Health Sciences, Kenya.	Community-Oriented Primary Care (COPC) Curriculum	Post program 30–60 minutes semi-structured interviews	Family medicine clerkship	8 family medicine trainee 5 faculty member	Identification and implement community-based health care interventions by family medicine postgraduate to guide in making a community diagnosis, SDH in participatory research.	1 Year
4	Goroncy et al., 2020	The Christ Hospital/University of Cincinnati (TCH/UC), United States.	Competency-based Home Visit Curriculum	Post program survey and thematic analysis of reviewed reflections.	Family medicine residents	43 residents completed survey 34 completed reflections	First year home visit: focus on modelling Second year home visits: continuity patients with faculty precipitators observing- physical and mental health of homebound older patients- overlooked population. Third year home visits: continuity of patients with focus on incorporating into practise.	4–6-week visits, one visits every 4–6 weeks in three years
5	Gard et al., 2020	Northwestern University Feinberg School of Medicine and the McGaw Medical Centre, United States.	Competence at Identifying and Addressing SDH.	Post program survey	Family medicine & internal medicine	95 internal medicine 34 family medicine	Identifying challenges to optimal health care that affect patients of low socioeconomic status, discussing these challenges during patients’ routine office visits, referring patients to resources within North-western Medicine to address the challenges; and “referring patients to local community resources.	2 Months
6	Jacobs et al.,2019	Saint Louis University Family Medicine Residency, United States.	Longitudinal Underserved Community Curriculum	Pre and post learning evaluation	Family medicine & psychiatry	22 residents completed program and evaluation pre,post and follow up	Workshops included conversations with patients, visits to community organizations, introduction to resources, hands-on clinical workshops, fieldwork, short didactic sessions, and awareness raising exercises.	3 Year curriculum including 12 monthly day-long community health workshop during year 2 focusing on health disparities
7	Ramadurai et al.,2021	University of Colorado, United States.	Case-based Curriculum Integrating SDH with Critical Care Topics	Pre and post program survey	Internal medicine Emergency medicine Family medicine residents	32 completed program 27 pre-program evaluation 11 post program evaluation	Social risk: housing instability, food insecurity, transportation problems, utility help needs, and interpersonal safety.	3 Years curriculum, including weekly curricula sessions, each session 30 minutes during the 4-week medical intensive care unit rotation
8	Knox et al.,2018	Aurora Family Medicine Residency Milwaukee, Wisconsin, United States.	Community Health, Advocacy, and Managing Populations	Thematic analysis of structured group and individual interviews	Internal medicine residents	20 in year one and 16 in year two completed program 16 completed evaluation	longitudinal curriculum for family medicine residents designed to integrate tools and skill building in community health, population health and management, advocacy, and health disparities into clinical practice	3 Years with combination of block mandatory rotations with longitudinal elective experience.
9	Christmas et al., 2020	Johns Hopkins Bayview Medical Centre, United States	Patient Care Centred Curriculum	Post rotation evaluation	Family medicine residents	150 completed program 94 completed evaluation	This curriculum includes a restructured admission history form, preference for bedside rounding, conducting phone calls to outpatient providers, and following up with patients after discharge	3 Years residency program with 2–8 weeks on intervention during training. Residents rotate on all 4 general medicine team during training for a minimum of 4 weeks.
10	Morrison et al., 2021	Johns Hopkins All Children’s Hospital Centre for Simulation, United States	Simulation-based SDH Training	Post program, survey, group discussions and projects.	Internal medicine residents	48 completed program 39 completed post program evaluation 13 completed follow up evaluation	The contents of the curriculum were derived from The National Academies of Sciences, Engineering and Medicine guidance.	1 Year with 45- minutes didactic base training.
11	Mullett et al., 2022	Department of Paediatrics, University of Washington, United States.	INCLUDE	Pre and post program survey and post-program reflection	Paediatric residents	109 completed pre-program evaluation 323 completed post program evaluation	1.Skill building training (10 hrs) 2.Didactics (15 hrs): during lunch time with multidisciplinary expertise + topics in clinic care, research and advocacy. 3.Small group discussion (3 hrs): evening book club discussion and morning reports for patient’s health equity	Longitudinal curriculum for 12 months with weekly didactics, interactive group discussions and journal clubs
12	Traba et al.,2021	The Rutgers New Jersey Medical School in Newark, New Jersey, United states	Health Equity Curriculum	Post exercise anonymous survey	Paediatric residents	75 completed program 61 completed evaluation	Curriculum content includes access to care, food insecurity, human trafficking, immigrant health, LGBT health, race and ethnicity, and women’s health.	Eight rotations (virtual interactive budgeting exercise 1.5–2 hours during the mandatory didactic 4 months rotation.
13	Balighian et al., 2020	Johns Hopkins University Paediatric and Medicine, United States.	Posthospitalization Home Visit Curriculum	Post- home surveys	Paediatric clerkship	31 completed program 29 completed evaluation	Home visit curriculum allowing residents to experience SDH first-hand by taking a metaphorical walk in their patients’ shoes, offering insights into their homes, families, neighbourhoods, streets, schools, grocery stores, and play spaces (or lack thereof), to provide a more practical understanding of what their daily life entails and how health care may be delivered at home.	4 Weeks (30 minutes didactic lessons on home visit at the beginning of the 4-week rotation during Y1 and Y2 of residency.
14	Tschudy et al.,2013	Johns Hopkins Hospital, United States.	Health Begins at Home	Pre and post evaluation, end of residency surveys	Paediatric residents	76 completed program 50 completed evaluation	Program based in the theoretical framework of social learning theory and the health belief model. Components of the module included discussion of the process of home visitation and social determinants of health using the communities surrounding.	One Year curriculum (1 hr. educational module, home visit and post home visit debriefing, 30 days pre-home visit survey before the educational module)
15	Sufrin et al., 2012	San Francisco County Jail, United States.	An Ambulatory Care rotation at County Jail	Post program online evaluation	First year obstetrics and gynaecology residents	9 completed program and evaluation	Pre session knowledge of the San Francisco County Jail guidelines, weekly reading, discussions regarding various topics ex: incarcerated women, and sociological analyses	One-half day per week during the 6 Weeks ambulatory care block rotation
16	O’Toole et al., 2012	Cincinnati Children’s Hospital Medical Centre (CCHMC), United States.	Clinic-Based Social and Legal Resources program	Pre and post online questioner	Second- and Third-year Paediatric resident Combined medicine & paediatric residents	46 completed program 40 completed evaluation	A novel screening tool for SDH related topic as food insecurity, housing, public benefits, depression, and domestic violence	5 Months
17	Lazow et al., 2019	Cincinnati Children’s Hospital Medical, USA	A virtual Neighbourhood Tour Curriculum	Post program online questioner	Paediatric residents	22 completed program 19 completed evaluation	1.Simulated online cases, to detect various aspects of SDH, and showing empathy 2. Simulated videos (one month after the virtual tour) are recorded (max 3 min) and uploaded to the online educational website 3. open-ended questions integrated with each video.	3 Online simulations (4 hour- one half day completed during advocacy rotation)
18	Lax et al., 2019	Children’s Hospital at Montefiore (CHAM), United States.	Three-Tiered Advocacy Curriculum	pre and post program survey	Paediatric residents & interns	138 completed pre-program evaluation 110 completed post program evaluation	Workshop1: introduction to advocacy, with small group case-based discussion IHELLP model (Income sources and benefits, Housing, Education, Legal Issues (including immigration), Literacy, and Parenting/ Psychosocial.) developed by the National Centre for Medical-Legal partnership. On an individual level and on community level and on a legislative level 2. Workshop 2: introduction part2: reinforcement of the concept by recognising the SDH impact on health outcomes 3. workshop 3: income and general benefits 4. workshop 4: housing and education 5. workshop 5: legal literacy and parenting 6. workshop 6: legislative advocacy 7. Lobby day to learn about the current legislations.	9 Months
19	Real et al., 2016	Cincinnati Children’s Hospital Medical Centre (CCHMC), United States.	Neighbourhood-based Curriculum	post evaluation	Paediatric residents	37 completed program 37 residents completed evaluation 148 caregiver completed evaluation	Topics included addressing housing problems (e.g., referral to medical-legal partnership), obtaining healthy food (i.e., food pantry locations, free meal programs), locating safe places to play (i.e., park locations, recreation centres), locating pharmacies (i.e., mapping neighbourhoods), and assisting families with transportation (i.e., public transportation, insurance-sponsored programs).	Two weeks advocacy, a year of continuity clinic experience 3–30 minutes small teaching modules separated by one month
20	Lochner et al., 2018	University of Wisconsin, Department of Family Medicine, United States.	Three Community Health Responsibilities for Family Doctors	Annual post program survey	All family medicine residents	323 faculty &120 residents completed program & evaluation	Understanding the difference between health and the health care system, (2) leading the way to an equitable, affordable health care system, and (3) being a community partner	3-year program at four continuity clinic sites in Dane County, Wisconsin
21	Schmidt et al., 2017	Emory University School of Medicine, United States.	Primary Care Centre Ambulatory Rotation	Pre-program survey and post program reflection	Second- and third-year Internal medicine residents	39 completed program 19 completed evaluation	1.Introductory workshops to discuss social disparities and the way to assess and address 2. Week 1: Identifying high risk patient, assess their social needs and creating management plan. 3. Week 2: Meet social worker or pharmacist to assess the patient 4. Week 3: Choose one of the patient social barriers and work in a small group to assess and create a plan. 5. Week 4: 45 min reflective workshop and demonstration of the facilitators.	Four weeks
22	Connors et al., 2022	University of Alberta, Canada	Impact of Social Paediatrics rotation on Residents’ understanding of SDH	Post program reflection and interviews	Paediatric residents second year	35 completed program and evaluation	Self-scheduled placement by the residents following a list of the placement to covered.	Four weeks
23	Real et al., 2017	Cincinnati Children’s Hospital, USA	Geomedicine Curriculum	Post- program questionnaire and caregiver’s interview	Paediatric residents first year	44 completed program and evaluation	The curriculum aimed for both intern and senior resident: including: 1. Familiarity, understanding, by free store food bank experience, jobs and family visit, neighbourhood tour. 2. Knowledge of neighbourhoods and resources though online games, brief lectures, and introduction of online resources. 3. Skills at providing neighbourhood based anticipatory guidance by group discussion and case-based simulation.	2 Weeks spiral curriculum through advocacy rotation, 3–30 minutes small teaching modules occurring before the start of continuity clinics
24	Lazow et al., 2018	Cincinnati Children’s Hospital Medical CCHMC), United States	Interactive Virtual Tour of a Local Impoverished neighbourhood using Innovative Technology	Pre and post survey	Paediatric residents and fellows	21 completed virtual tour 25 completed self-assessment 46 completed evaluation	The virtual tour application was designed based on the principles of cognitive load theory, which describes strategies to decrease the cognitive load imposed on learners’ limited capacity working memory to ensure sufficient processing of new information into long-term memory. The program combined visual and auditory modalities and designated specified pauses to allow learners time to self-explain and process complex information in a stepwise manner.	8 virtual neighbourhood tour, meeting with 14 community leaders and patients in one year.

	Educational Methods Applied			Program Evaluation			Quality Assessment	
	Didactic	Participatory Learning	Community Placement	Affective	Knowledge	Performance	Quality Assessment 1	Quality Assessment 2
1		X			X		9	9
2	X	X	X			X	9	9
3		X			X		9	10
4	X	X	X	X	X		9	10
5		X	X	X			11.5	11.5
6	X	X	X		X		10.5	11
7	X	X	X		X		12	11.5
8		X			X		9.5	9
9		X	X		X		10.5	10
10	X	X	X		X		10.5	10
11	X	X		X	X		11.5	12
12	X	X			X		11	10
13	X	X	X		X		11	10
14	X	X	X		X		10.5	9.5
15	X	X	X		X		12	11
16		X	X	X			9	9.5
17		X	X		X		12	12.5
18	X	X			X	X	10	10.5
19	X	X	X	X	X		11.5	11
20	X	X	X	X	X		9.5	10.5
21		X	X	X	X		10	12
22			X	X	X		11	11.5
23	X	X	X	X	X		9.5	10.5
24		X			X		10	10

The structure of the programs related to SDH varied across a range of thematic areas. A total of five courses had a focus on home visits and different community healthcare interventions [23, 30, 31, 40, 41], while another set of 10 programs was in the form of case-based workshops on a variety of topics such as prison healthcare, housing issues locating pharmacies and follow-up of patients after discharge [24–26, 28, 29, 32, 34, 39, 43, 45] Lastly, nine programs focused on health advocacy topics, such as opportunities to integrate SDH at community health clinics, housing, education, and legal issues, integration of health disparities to clinical practices and equity, diversity, and inclusion [22, 27, 33–38, 44].

### Programs presentation methods

The approach to presenting the graduate SDH training and learning activities varied. All the programs used participatory learning, “where the learners are actively participating instead of being passive listeners,” as an educational strategy in combination with other teaching modalities. Eleven programs combined participatory learning with community placement and didactic teaching [23–25, 28, 31, 33, 34, 36, 40–42]. Another six programs relied on a participatory approach, with community placement and no formal lectures [27, 35, 36, 43–45]. Three programs integrated didactic teaching and a participatory approach with no community engagement [29, 37, 38]. Another set of four programs included participatory learning only, requiring participant engagement, such as information gathering, group discussions, and activities [22, 26, 32, 39].

### Evaluation of the graduate SDH programs

All the reviewed programs (*n* = 24) had an evaluation component in their curriculum. Six programs used pre- and post-learning evaluation surveys [24, 25, 30, 32, 35, 38], while 11 programs used only post-learning evaluation surveys [22, 27, 28, 31, 36, 37, 39–41, 44, 45]. Three programs used thematic analysis of participants’ written reflections and interviews [26, 34, 44]. One program used both post-course interviews and participants’ reflections analysis [23]. One program combined pre and post-surveys with participants’ reflections [29]. Another program used pre-surveys and post-course reflections [43].Only one program evaluated the participants and the patient’s primary guardians’ views [33].

Five programs evaluated the participants’ affective learning, including their awareness, interest, and empathy combined with their level of knowledge regarding the SDH within the local context [23, 29, 31, 42, 44]. Another three programs used affective learning assessment solely [33, 35, 41]. One program adopted a comprehensive assessment on the three levels, including participants’ attitudes, knowledge, and performance [43]. Another program incorporated knowledge and performance as an evaluation tool [38], and one used the candidate’s performance as the main evaluation aspect [34]. Additionally, 13 programs only used the participants’ knowledge level as an evaluation indicator [22, 24–28, 30, 32, 37, 39–41, 45]..

## Discussion

This work details a scoping review of literature relating to incorporating the SDH in graduate medical training curricula. Notably, of a total of 24 included articles, 22 programs were implemented in the USA medical schools [23–43, 45], with one program in Canada [44] and only one from a low- and middle-income country (Kenya) [22]. The evaluation of the programs varied on different levels; most programs performed post-learning evaluation only for the participants, and only one program added the patient’s perspective on the quality of service provided. The evaluation modules used need more clarity in reporting. The programs with extended training over the years reported a more favorable impact on the knowledge and the participant’s skills regarding SDH concepts. Participants favored training programs that blinded academic knowledge with community placement.

Paediatric training programs took the lead in training healthcare professionals in SDH. Other specialties, such as internal medicine, family medicine, and psychiatry, needed to be more proactive in integrating the SDH into their curriculum. Incorporating SDH concepts for all healthcare training is essential for weaving socially accountable healthcare into healthcare systems [46]..

Participants rated the SDH programs with a multi-year longitudinal structure highly. This finding agrees with other studies suggesting that spiral training programs improve trainees’ community integration, mentorship, confidence, knowledge in evidence-based medicine, patient-centred care, and reflective practice [47–50].. Our study found heterogeneity in each program’s content, as SDH factors can differ from one geographical location to another. The WHO study states that educators should apply a local context approach to tackle this issue [51]..

All the programs’ teaching strategies involved the participants in the teaching process, so-called “participatory learning.” The programs integrated academic knowledge with community placement and significantly impacted the comprehension of SDH concepts and their application in real-life situations. These findings correlate with studies emphasizing that combining theoretical learning with community engagement will enhance participants’ ability to cultivate an understanding of the core principles of the taught subject [52–57]..

Finally, most programs evaluated the participants’ knowledge level and confidence in recognizing SDH-related factors pre- and post, or post-program only. The reported evaluation outcomes included improved knowledge, awareness, and trust in dealing with diverse and underserved communities. Only one program interviewed the patients’ guardians and evaluated the care received by the trained physician [33]. This finding highlights a gap in program evaluation and the need to identify standardized criteria to monitor the success of SDH teaching in postgraduate curricula [58]..

### Study limitations and strengths

The number of published articles demonstrating the implementation of SDH training in postgraduate programs is limited. This limitation is likely a significant under-representation of the innovation and scope of SDH integration into postgraduate curricula and again highlights the need for more high-quality literature assessing the effective incorporation, delivery, and assessment of SDH competencies. The scope of articles available in English primarily limited our study. The study focused on the programs including SDH teaching as a separate module not included with public health or global health. Our study is constrained by the unavailability of data from specific databases, which has restricted the scope of our research. Despite these limitations, our study has several strengths. Our study represents a pioneering effort in the field by conducting a comprehensive analysis of integrating SDH into graduate medical training programs. The significance of this research lies in its ability to shed light on the current state of these programs and identify critical areas for improvement. This study displays the heterogeneity of evaluation for such training programs and the deficiency in following the downstream impact of this training on patients’ health. These findings further support questions raised by medical education experts such as Sharma et al. (2018), who explained the importance of SDH teaching and the role of educators and training institutions yet criticized the focus on integration rather than evaluation [59]..

#### Implications for practice and future research

Our review has identified several future research implications; there needs to be more representation of the published literature about the topic in general and from low- and middle-income countries. The different expression of the SDH training programs by the developed countries’ training institutions may be because of the influence of The Accreditation Council for Graduate Medical Education (ACGME). The ACGME approves complete and independent medical education programs in the United States and Canada. The ACGME standards include addressing health equity and enhancing cultural competency through the taught curriculum of the accredited graduate program, which compels medical institutions to integrate SDH into their curricula [60, 61]. This shows the critical influence accrediting bodies have on the content of medical curricula. As the United Nations (UN) stated in 2015, low- and middle-income countries face triple the burden of health issues and, therefore, creating a well-trained healthcare force and robust health system performance will decrease social disparities [62, 63].

## Conclusion

Integrating SDH into graduate medical education curricula is a dynamic and evolving area of research and practice. While the literature highlights the growing recognition of the importance of SDH education, it also reveals gaps in standardized curriculum development, assessment strategies, and long-term evaluation. Providing a multi-level structure approach for the methodology, implementation, and evaluation of SDH training programs will allow training bodies and institutions to integrate SDH concepts more effectively and produce a transparent blueprint for others to follow. Addressing these gaps will ensure that medical graduates are prepared to overcome complex SDH in healthcare.

## Data Availability

Data are available upon request by the corresponding author.
